# A new and widely distributed species of *Ichthyurus* Westwood, 1848 (Cantharidae, Chauliognathinae, Ichthyurini) from China

**DOI:** 10.3897/BDJ.12.e131829

**Published:** 2024-09-16

**Authors:** Hanqing Lin, Haoyu Liu, Xingke Yang, Yuxia Yang

**Affiliations:** 1 Key Laboratory of Zoological Systematics and Application, School of Life Science, Institute of Life Science and Green Development, Hebei University, Baoding, China Key Laboratory of Zoological Systematics and Application, School of Life Science, Institute of Life Science and Green Development, Hebei University Baoding China; 2 Hebei Basic Science Center for Biotic Interaction, Hebei University, Baoding, China Hebei Basic Science Center for Biotic Interaction, Hebei University Baoding China; 3 Key Laboratory of Zoological Systematics and Evolution, Institute of Zoology, Chinese Academy of Sciences, Beijing, China Key Laboratory of Zoological Systematics and Evolution, Institute of Zoology, Chinese Academy of Sciences Beijing China

**Keywords:** *Ichthyurus*, Alpha taxonomy, new species, soldier beetles, East Asia

## Abstract

**Background:**

The genus *Ichthyurus* Westwood, 1848 is a large cantharid group consisting of approximately 200 species worldwide, with only 10 species hitherto found in China. Despite its expansive area, the Chinese fauna has historically received little attention from specialists, leading to a lack of knowledge even about some common *Ichthyurus* species in this region.

**New information:**

A new species of *Ichthyurus* Westwood, 1848 is described under the name of *I.longulus* sp. nov., which is widely distributed in mainland China, including Shannxi, Gansu, Hubei, Chongqing, Guizhou and Guangxi. Although there are some variations in the pronotum colouration within the species, this new species could be easily distinguished from all others of *Ichthyurus* by the large-sized body, uniformly black elytra, mesotibiae each with an apical spur in male, terminal abdominal tergite of male with long and cylindrical lateral projections that are about 3/5 the length of the tergite, terminal abdominal ventrite of male saddle-shaped and deeply cleft in middle of apical 2/3 part and aedeagus with a long setifore extension that is as long as the parameres. The habitus, terminal abdominal ventrite and tergites and genitalia of both sexes are illustrated. In addition, a distribution map of this species and a list of the *Ichthyurus* species from mainland China are provided.

## Introduction

The genus *Ichthyurus* Westwood, 1848 represents the largest taxonomic group within the tribe Ichthyurini of soldier beetles, encompassing approximately 200 species distributed worldwide (Delkeskamp 1977, Brancucci 1983, Kazantsev and Brancucci 2007, Brancucci 2009, Li et al. 2014, Li and Gao 2015). China, with an expansive area of 9,600,000 km^2^, constitutes a significant component part of the global fauna (Zhang 1999). Despite its vast geographical extent, this country exhibits a limited number of known species belonging to *Ichthyurus*. Until now, there are a total of 10 Chinese species, amongst which four species are endemic to Taiwan Island (Brancucci 1983, Satô et al. 2014) and the others restricted to mainland China (Delkeskamp 1977, Kazantsev and Brancucci 2007, Jiang and Yang 2023). For the latter species, most of them were described almost one century ago (Gestro 1892, Gestro 1906, Pic 1924, Pic 1926, Pic 1928).

In our study, we assembled a large amount of *Ichthyurus* material from mainland China. After conducting identification and comparison, also referring to relevant literature, as well as examining hundreds of types, we have discovered dozens of new species, which are being or will be described in our forthcoming publications. It is worth noting that the Chinese fauna of *Ichthyurus* has historically received little attention from specialists, so that even some common species remain unknown in this region. In the present study, we are going to describe one new species that is widely distributed in central and western China.

## Materials and methods

The studied materials are preserved in the following collections:

- **MGI** Museo Civico di Storia Naturale “Giacomo Doria”, Genova, Italy;
- **MHBU** Museum of Hebei University, Baoding, China;
- **MNHN** Muséum national d’Histoire naturelle, Paris, France;
- **IZAS** Institute of Zoology, Chinese Academy of Sciences, Beijing, China;
- **SNUC** Insect Collection of Shanghai Normal University, Shanghai, China;
- **GUGC** Institute of Entomology, Guizhou University, Guiyang, China.

The specimens were initially softened in water, followed by the dissection of the genitalia and genital segments of both sexes. Subsequently, the male genitalia were immersed in a 10% sodium hydroxide (NaOH) solution and then placed in a metal bath warmed to 90℃ for one minute. After that, the clean male genitalia were examined and photographed in glycerol and then affixed to a paper card for permanent preservation. The female genitalia were stained with haematoxylin, examined in 75% alcohol and preserved in glycerol. For species with multiple distributions, each distribution is compared through dissections of both female and male specimens. In cases where any damage occurred during dissection, additional specimens would be treated as necessary.

The habitus images were captured using a Canon EOS 80D digital camera and others with a Leica M205A stereomicroscope. Multiple layers were stacked using Helicon Focus 7 and post-processing of the images and the measurements was conducted in Photoshop 2020. The distribution map was prepared by ArcMap 10.8 and edited in Adobe Photoshop 2020.

For the specimens, quotation marks are used if their original labels are written in English. All labels written in Chinese are transliterated into English. Morphological terminology in this study follows Brancucci (Brancucci 1980). The French term “bride tergale” is translated into English as “tergal flange”. Body length was measured from the front of head to the apices of the lateral projections of the terminal abdominal tergite and width at elytral humeri. Interocular distance was measured at the minimal point between eyes and diameter of an eye at its maximal point.

## Taxon treatments

### Ichthyurus longulus

Y. Yang, Lin & Liu sp. nov.

133C3ECD-A23A-5F86-AB57-DC5B1948B466

3085196D-1BCA-4449-B52A-0F991467B41E

#### Materials

- **Type status:** Holotype. **Occurrence:** recordedBy: Hanqing Lin; sex: 1 male; occurrenceID: 68041DAD-2F34-50A7-8275-90B683A81B1C; **Location:** country: China; county: Chongqing; municipality: Jinfoshan, Sanquan, Qishutang; verbatimElevation: 1288 m; verbatimLatitude: 29.05684°N; verbatimLongitude: 107.16292°E; **Event:** year: 2024; month: 5; day: 14; **Record Level:** institutionID: Museum of Hebei University; institutionCode: MHBU
- **Type status:** Paratype. **Occurrence:** recordedBy: Hanqing Lin; sex: 3 males, 3 females; occurrenceID: EEE9D361-7CB3-5222-8F85-210B28DA1388; **Location:** country: China; county: Chongqing; municipality: Jinfoshan, Sanquan, Qishutang; verbatimElevation: 1288 m; verbatimLatitude: 29.05684°N; verbatimLongitude: 107.16292°E; **Event:** year: 2024; month: 5; day: 14; **Record Level:** institutionID: Museum of Hebei University; institutionCode: MHBU
- **Type status:** Paratype. **Occurrence:** recordedBy: Jialing Chen; sex: 1 male, 1 female; occurrenceID: 5897DA38-C9B0-5630-8F0A-39C0903CCC2A; **Location:** country: China; county: Chongqing; municipality: Jinfoshan, Sanquan, Nanmuwan; verbatimElevation: 1304 m; verbatimLatitude: 29.05471°N; verbatimLongitude: 107.17426°E; **Event:** year: 2024; month: 5; day: 13; **Record Level:** institutionID: Museum of Hebei University; institutionCode: MHBU
- **Type status:** Paratype. **Occurrence:** recordedBy: Liang Zhang; sex: 1 male; occurrenceID: 29ADF61A-4BBD-5280-83CB-494132E08CEE; **Location:** country: China; county: Chongqing; municipality: Toudu, Dianchanggou; verbatimElevation: 902 m; verbatimLatitude: 29.01685°N; verbatimLongitude: 107.08325°E; **Event:** year: 2024; month: 5; day: 8; **Record Level:** institutionID: Museum of Hebei University; institutionCode: MHBU
- **Type status:** Paratype. **Occurrence:** recordedBy: Ping Wang; sex: 1 male; occurrenceID: 22CEEED2-BAAE-54C3-9CF1-035B3652E8D1; **Location:** country: China; county: Chongqing; municipality: Jinfoshan, Sanquan; verbatimElevation: 1303 m; verbatimLatitude: 29.05460°N; verbatimLongitude: 107.17426°E; **Event:** year: 2024; month: 5; day: 30; **Record Level:** institutionID: Museum of Hebei University; institutionCode: MHBU
- **Type status:** Paratype. **Occurrence:** recordedBy: Wangang Liu; sex: 1 male; occurrenceID: 2AB46BC0-2331-52F0-82BD-01D462B1D75C; **Location:** country: China; county: Chongqing; municipality: Jinfoshan; verbatimElevation: 1601 m; verbatimLatitude: 29.08333°N; verbatimLongitude: 107.18333°E; **Event:** year: 2010; month: 6; day: 13; **Record Level:** institutionID: Institute of Zoology, Chinese Academy of Sciences; institutionCode: IZAS
- **Type status:** Paratype. **Occurrence:** recordedBy: Caixia Yuan and Yushuang Liu; sex: 1 female; occurrenceID: B90C235A-94CA-58A8-B631-8EE288F1B222; **Location:** country: China; stateProvince: Shannxi; municipality: Ziyang, Maoba; verbatimElevation: 1303 m; verbatimLatitude: 29.05460°N; verbatimLongitude: 107.17426°E; **Event:** year: 2003; month: 7; day: 8; **Record Level:** institutionID: Museum of Hebei University; institutionCode: MHBU
- **Type status:** Paratype. **Occurrence:** recordedBy: Xingke Yang; sex: 1 female; occurrenceID: F518E424-6255-587C-8AAA-F5210046DAB0; **Location:** country: China; stateProvince: Gansu; municipality: Wenxian, Bikou, Bifenggou; verbatimElevation: 900‒1450 m; **Event:** year: 1998; month: 6; day: 25; **Record Level:** institutionID: Institute of Zoology, Chinese Academy of Sciences; institutionCode: IZAS
- **Type status:** Paratype. **Occurrence:** recordedBy: Tengfei Qiu; sex: 1 male; occurrenceID: 460A0351-0598-5D67-9DA4-40FC28750D7F; **Location:** country: China; stateProvince: Hubei; county: Yichang; municipality: Dalaoling; **Event:** year: 2015; month: 5; day: 6; **Record Level:** institutionID: Museum of Hebei University; institutionCode: MHBU
- **Type status:** Paratype. **Occurrence:** recordedBy: Xiujuan Yang; sex: 1 female; occurrenceID: 10FF9330-9638-5D0A-A343-8473E1E8F7BB; **Location:** country: China; stateProvince: Guangxi; county: Baise; municipality: Tianlin, Yaojiawan; verbatimElevation: 1200‒1400 m; **Event:** year: 2002; month: 4; day: 1; **Record Level:** institutionID: Museum of Hebei University; institutionCode: MHBU
- **Type status:** Paratype. **Occurrence:** recordedBy: Yang Yu; sex: 1 female; occurrenceID: C7A17B9C-FB3F-5CFF-98C5-06E41AE91381; **Location:** country: China; stateProvince: Guizhou; county: Daozhen; municipality: Yangxi, Qishiyan; **Event:** year: 2004; month: 4; day: 29; **Record Level:** institutionID: Museum of Hebei University; institutionCode: MHBU
- **Type status:** Paratype. **Occurrence:** recordedBy: Jiang, Jiang, Hu, Liu, & Zhang; sex: 1 female; occurrenceID: ADAA029C-424E-5ED0-9722-AE9C9B96FB76; **Location:** country: China; stateProvince: Guizhou; county: Libo; municipality: Maolan N. R., Bizuo; verbatimElevation: 587 m; verbatimLatitude: 25.28306°N; verbatimLongitude: 108.05500°E; **Event:** year: 2017; month: 4; day: 28; **Record Level:** institutionID: Insect Collection of Shanghai Normal University; institutionCode: SNUC
- **Type status:** Paratype. **Occurrence:** recordedBy: Jiang, Jiang, Hu, Liu, & Zhang; sex: 1 female; occurrenceID: 3719E996-959A-592A-82DE-DC6BBE61FF78; **Location:** country: China; stateProvince: Guizhou; county: Libo; municipality: Maolan N. R., Dongdai; verbatimElevation: 865 m; verbatimLatitude: 25.31417°N; verbatimLongitude: 107.95056°E; **Event:** year: 2017; month: 4; day: 23; **Record Level:** institutionID: Insect Collection of Shanghai Normal University; institutionCode: SNUC
- **Type status:** Paratype. **Occurrence:** recordedBy: Tianjun Liu; sex: 1 female; occurrenceID: 6764D079-A8F2-5FA2-8DEE-B9509B8F9858; **Location:** country: China; stateProvince: Guizhou; municipality: Jiangkou, Kuaichang; **Event:** year: 2021; month: 5; day: 28; **Record Level:** institutionID: Institute of Entomology, Guizhou University; institutionCode: GUGC
- **Type status:** Paratype. **Occurrence:** recordedBy: Tianjun Liu; sex: 1 female; occurrenceID: 3B3E03D5-DDFD-5B4D-9897-E5A7DC229F8B; **Location:** country: China; stateProvince: Guizhou; municipality: anjingshan, Jiangkou, Kuaichang, Mingjingshan; **Event:** year: 2021; month: 5; day: 26; **Record Level:** institutionID: Institute of Entomology, Guizhou University; institutionCode: GUGC

#### Description

Body length (both sexes): 13.5‒14.4 mm (13.5 mm in holotype); width at humeri (both sexes): 1.9‒2.0 mm (1.9 mm in holotype).

**Figure 1.**
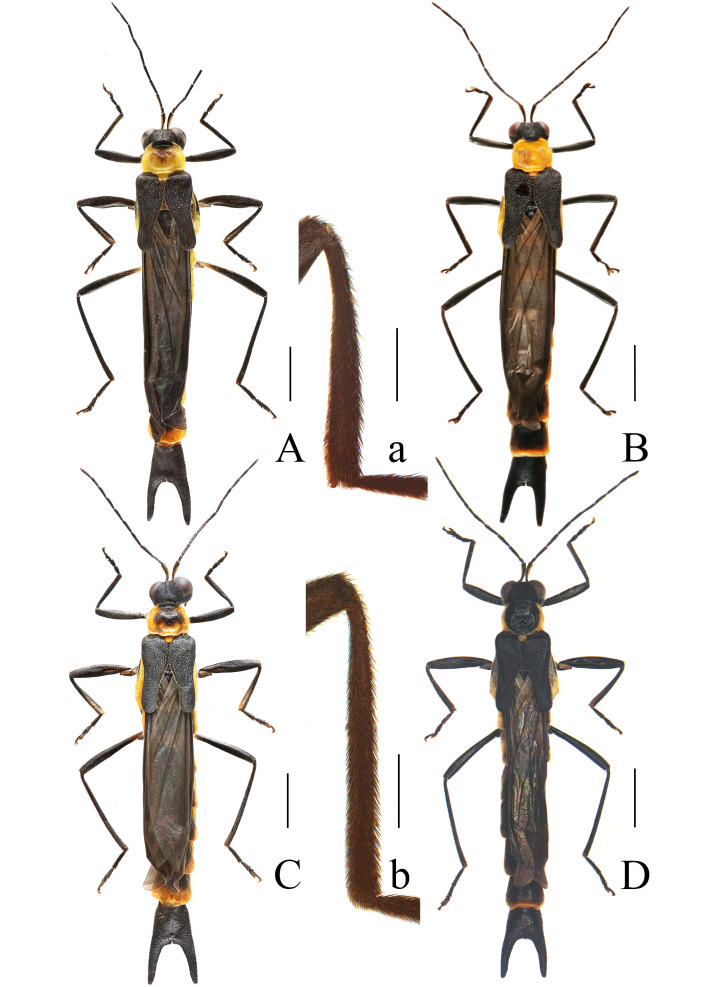
*Ichthyuruslongulus* sp. nov. (**A‒D, a** male; **b** female): **A‒D** habitus, dorsal view; **a‒b** right mesotibia, lateral view. Scale bars: A‒D: 2.0 mm, a‒b: 0.5 mm.

**Male** (Fig. 1A). Colouration. Body black, antennomere I yellow ventrally; prothorax yellow, pronotum with a small black marking at middle of anterior margin; metaventrite each side with a longitudinal yellow marking; metanepisternum and scutellum yellow; abdominal segments II‒VII narrowly yellow at lateral and posterior margins.

Eyes large, interocular distance about half of maximum eye diameter. Antennae reaching posterior margin of abdominal tergite III, antennomeres II about 1/3 of the length of I, III‒XI subequal in length and about 4.0 times longer than II.

Pronotum 1.1 times wider than long, anterior margin strongly arcuate and lateral margins slightly arcuate, posterior margin feebly bisinuate, anterior angles rounded and posterior angles nearly rectangular.

Leg slender, mesotibiae each present with a spur at inner apical angle (Fig. 1a).

Elytra 1.3 times longer than humeri width of conjoint elytra, twice longer than pronotum, with lateral margins sinuate, sutures moderately dehiscent at posterior 2/3 part, distance between sutures twice wider than width of an elytron, apices rounded.

**Figure 2.**
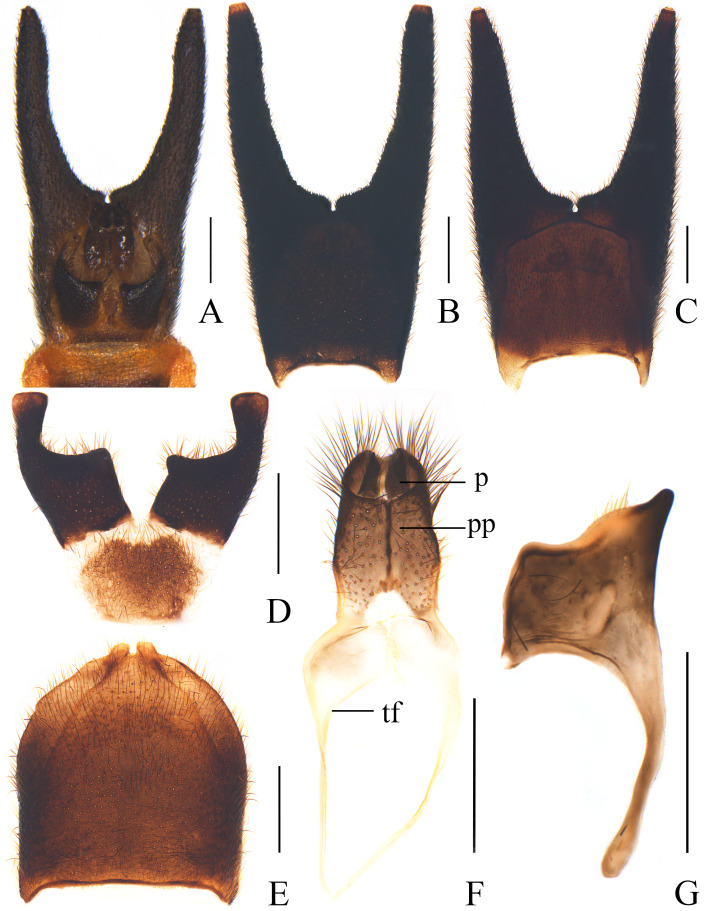
*Ichthyuruslongulus* sp. nov. (A, B, D, F, G male; C, E female): **A** genital segments in natural state, ventral view; **B‒C** terminal abdominal tergite, ventral view; **D‒E** terminal abdominal ventrite, ventral view; **F** proctiger and paraproct, ventral view; **G** abdominal sternite IX, ventral view. Abbreviations: p: proctiger; pp: paraproct; tf: tergal flange. Scale bars: **A‒F**: 0.5 mm, **G**: 0.2 mm.

Terminal abdominal tergite (or tergite VIII) (Fig. 2A and B) with lateral projections long and cylindrical, about 3/5 of the length of the tergite. Terminal abdominal ventrite (or sternite VIII) (Fig. 2D) widened apically, saddle-shaped and deeply cleft in middle of apical 2/3 part, with lateral margins feebly arcuate, inner apical angles largely and rectangularly emarginate, outer apical angles feebly widened and truncate at apices.

Abdominal sternite IX completely concealed underneath terminal ventrite (Fig. 2A), axe-like (Fig. 2G), with posterior right angle triangularly projecting posteriorly, posterior left angle rectangular, anterior right angle strongly and narrowly protruding anteriorly, rod-like and rounded at apex.

Proctiger (Fig. 2F) completely surrounded by paraproct, strongly sclerotised on both sides and almost membranous in middle, with lateral margins converging posteriorly. Paraproct (Fig. 2F) well-developed and tubular, feebly shrunk apically, shallowly emarginate in middle of dorso-posterior margin, present with a median longitudinal ridge on ventral side, surface coarsely punctate and covered with long and stout setae, tergal flange weakly sclerotised, strongly expanded at base and narrowly protruding apically.

Aedeagus (Fig. 4A‒D): left and right parameres both slender and nearly straight, subequal in length (Fig. 4A, D), rounded at apex; right paramere covered with a few short setae at outer side (Fig. 4B); setifore extension strongly sclerotised, nearly as long as parameres, with a cluster of long setae at apex (Fig. 4B and C); median lobe medium-length, about twice longer than parameres, apex rounded.

**Female** (Fig. 1B). Similar to males, but mesotibial spur absent (Fig. 1b), terminal abdominal tergite with lateral projections feebly stouter and horn-shaped, progressively thinned apically (Fig. 2C); terminal abdominal ventrite (Fig. 2E) parallel-sided and arcuately narrowed at posterior 1/3 part, present with a pair of small protuberances in middle of posterior margin.

Reproductive system (Fig. 4E‒F): coxites long and fused medially, styles thin and short; vagina elongate, with median oviduct situated in middle of ventral side; bursa copulatrix arising from apex of vagina, swollen and suddenly thinned apically; accessory gland opening at dorsal base of bursa copulatrix, long and slightly expanded at apical 3/4 part; a spermatheca in the form of two short canals and arising from ventro-basal part of bursa copulatrix.

**Figure 3.**
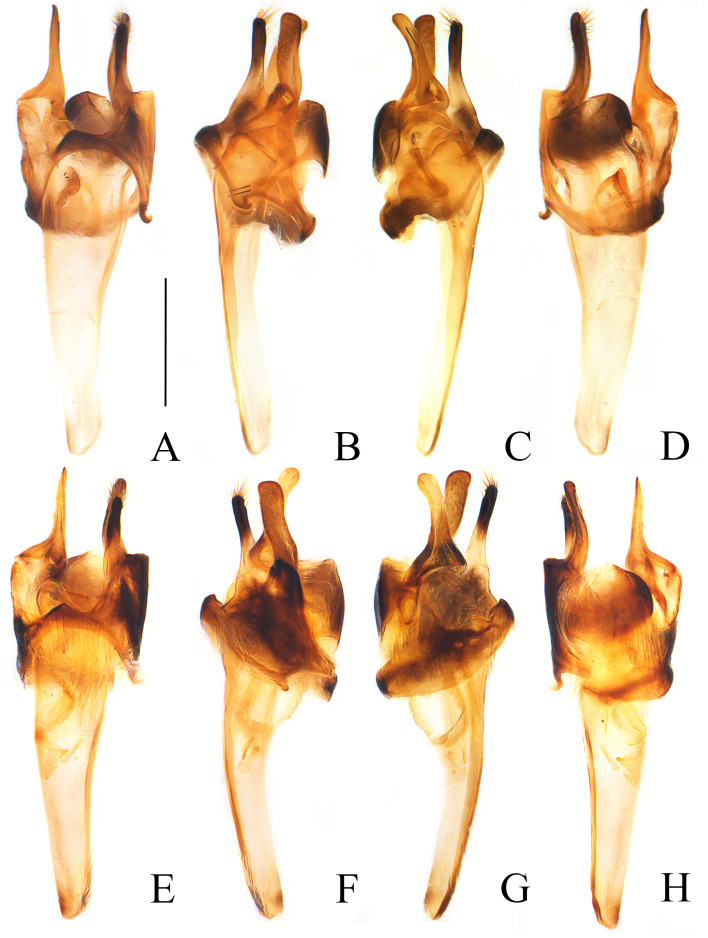
Aedeagi of *Ichthyuruslongulus* sp. nov.: **A‒D** paratype with a large black marking on pronotum; **E‒H** paratype with almost black pronotum. **A, E** dorsal view; **B, F** left-lateral view; **C, G** right-lateral view; **D, H** ventral view. Scale bars: 0.5 mm.

**Variation within type series.** Sometimes pronotum with a large black marking at anterior half part of the disc (Fig. 1C) or almost black with narrow yellow lateral margins (Fig. 1D). Their aedeagi (Fig. 3) same to the holotype.

#### Diagnosis

**Figure 4.**
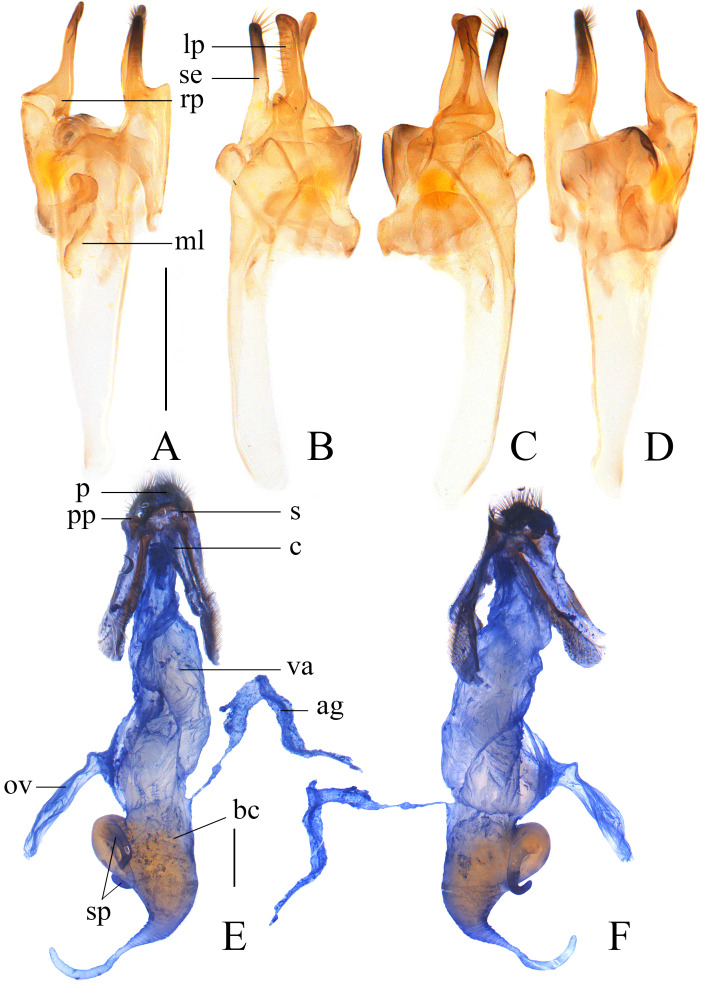
*Ichthyuruslongulus* sp. nov.: **A‒D** aedeagus (**A** dorsal view; **B** left-lateral view; **C** right-lateral view; **D** ventral view); **E‒F** female reproductive system (**E** ventro-lateral view; **F** dorsal-lateral view). Abbreviations: lp: left paramere; rp: right paramere; se: setifore extension; ml: median lobe; p: proctiger; pp: paraproct; s: style; c: coxites; va: vagina; ov: median oviduct; bc: bursa copulatrix; sp: spermatheca; ag: accessory gland. Scale bars: **A‒D** 0.5 mm; **E, F** 1.0 mm.

It can be easily distinguished from all Chinese species by the uniformly black elytra (Fig. 1), as opposed to the bicoloured and mixed with black and yellow in others. Meanwhile, each mesotibia is present with an apical spur in male (Fig. 1a), while absent in the other species or, at most, each protibia with an apical spine in *I.vandepolli* Gestro, 1892. Additionally, its terminal abdominal tergite is characterised by long lateral projections, approximately 3/5 of the length of the tergite (Fig. 2A‒B), whereas in other species, it is, at most, half as long. Furthermore, its aedeagus bears a long setifore extension nearly equal in length to the parameres (Fig. 4A and D), unlike other species where it is much shorter than the parameres. Moreover, it can be readily differentiated from *I.davidi* Gestro, 1892 and *I.vandepolli* by its slender legs in male (Fig. 1A, C and D) (while meso- or profemora are at least moderately expanded in the latter species) and from *I.bourgeoisi* 1892 and *I.savioi* Pic, 1928 by its terminal abdominal tergite with simple lateral projections in male (Fig. 2A‒B) (as opposed to being excavated ventrally in the latter species).

#### Etymology

The specific name is derived from the Latin *longus* (long), referring to its long setifore extension.

#### Distribution

**Figure 5.**
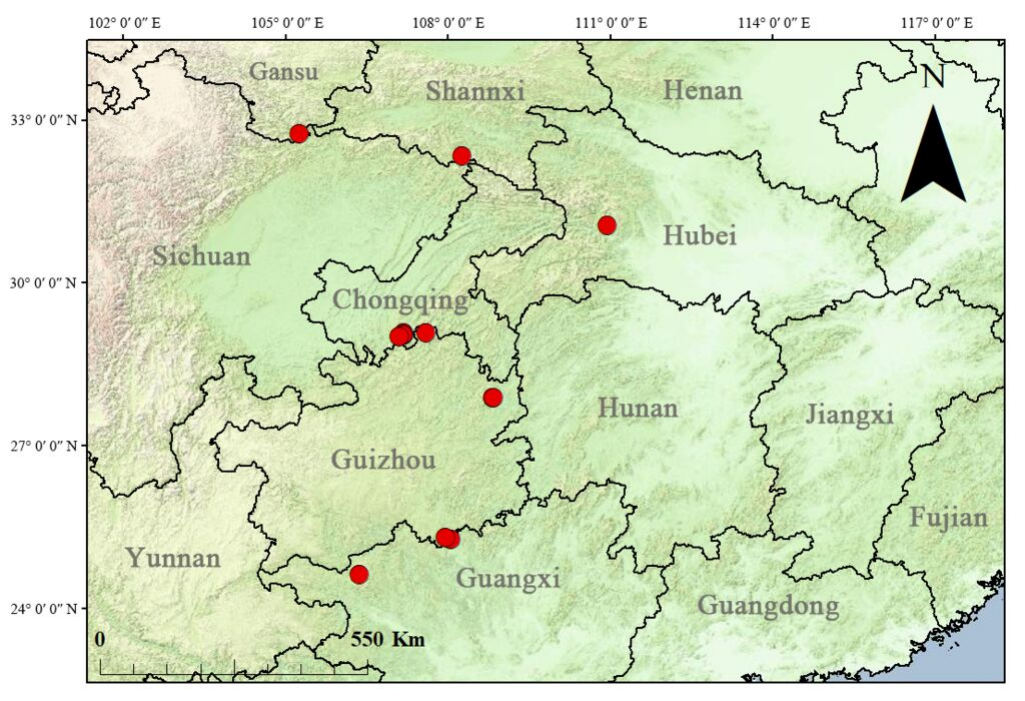
Distribution map of *Ichthyuruslongulus* sp. nov.

China (Chongqing, Shannxi, Gansu, Hubei, Guangxi, Guizhou) (Fig. 5).

## Checklists

### A list of the Ichthyurus species from mainland China

#### Ichthyurus bourgeoisi

Gestro, 1892

ED526D9C-B3D3-577C-8D3C-BC31653912B4

##### Distribution

W. China

##### Notes

Citation: Gestro (1892): 1023; Gestro (1906): 284; Delkeskamp (1977): 470; Kazantsev and Brancucci (2007): 297; Jiang and Yang (2023): 244. Type locality: Cina, W. Szunden [unclear locality, China]; type depository: MGI.

#### Ichthyurus davidi

Gestro, 1892

B243DA4A-4F19-570B-82DE-6A8AA20F74F0

##### Distribution

China (Jiangxi)

##### Notes

Citation: Gestro (1892): 1038; Delkeskamp (1977): 470; Kazantsev and Brancucci (2007): 297; Jiang and Yang (2023): 244. Type locality: Kian-Si [Jiangxi, China]; type depository: MGI.

#### Ichthyurus pieli

Pic, 1924

39C30844-87BE-5455-B9EB-9A48FB6446A8

##### Distribution

China (Shanghai)

##### Notes

Citation:Pic (1924): 89; Delkeskamp (1977): 472; Kazantsev and Brancucci (2007): 297: Jiang and Yang (2023):244. Type locality: Shanghai, China; type depository: MNHN.

#### Ichthyurus savioi

Pic, 1928

6BA85D67-ABEA-5247-A4EF-6CB7D16C9A89

##### Distribution

China (Anhui)

##### Notes

Citation: Pic (1928): 14; Delkeskamp (1977): 473; Kazantsev and Brancucci (2007): 297; Jiang and Yang (2023): 244. Type locality: Yue-Wan K. [Anhui, China]; type depository: MNHN.

#### Ichthyurus senensis

Pic, 1926

540829D6-A6BD-5D73-962C-F18B67F5D5A4

##### Distribution

China (Yunnan)

##### Notes

Citation: Pic (1926): 5; Delkeskamp (1977): 473; Jiang and Yang (2023): 244. Type locality: Yunnan, China; type depository: MNHN.

Remark. This species was missing in the Palaearctic Catalogue (Kazantsev and Brancucci 2007).

#### Ichthyurus vandepolli

Gestro, 1892

80357826-1D89-5F0B-BD75-A16CE9387080

##### Distribution

S. China

##### Notes

Citation: Gestro (1892): 1029; Gestro (1906): 283; Delkeskamp 1977: 473; Kazantsev and Brancucci (2007): 297; Jiang and Yang (2023): 244. Type locality: Cina bor., Fostune [unclear locality, China]; type depository: MGI.

#### Ichthyurus longulus

Y. Yang, Lin & Liu, sp. nov.

7D992877-2B0C-5F76-B93E-10C6D40E7C3E

3085196D-1BCA-4449-B52A-0F991467B41E

##### Distribution

China (Chongqing, Shannxi, Gansu, Hubei, Guizhou, Guangxi)

##### Notes

Citation: *Ichthyuruslongulus* Y. Yang, Lin & Liu, sp. nov. Type locality: Chongqing, China; type depository: MHBU.

## Discussion

*Ichthyuruslongulus* sp. nov. is a commonly found species with a wide distribution in central and western China (Fig. 5). It could be easily distinguished from all other Chinese species by the uniformly black elytra (Fig. 1), while others have bicoloured or mixed black with yellow elytra. There is some variation in the colouration of the pronotum (Fig. 1) within this species, but this variation occurs amongst different individuals in the same locality (China, Chongqing: Nanchuan, Jinfoshan National Natural Reserve). Further examination of their aedeagi (Fig. 4A‒D and Fig. 3) reveals that no differences can be found amongst them, confirming that they are conspecific. Although there is variability in the colouration of the pronotum, its elytra are consistently black in all individuals.

Furthermore, its separate status could be verified by combination of some other characters: mesotibiae each present with an apical spur in male (Fig. 1a), while absent in all others; terminal abdominal tergite of male with a pair of long lateral projections that are 3/5 length of the tergite (Fig. 1A, C and D), while short and less than 1/2 the length of the tergite in others; aedeagus with a long setifore extension that is nearly as long as parameres (Fig. 4A‒D and Fig. 3), while much shorter and, at most, 1/2 length of parameres.

In our study of *Ichthyurus*, we have adopted the concept of a species group (e.g. Okushima (2005), Švihla (2005), Yang et al. (2019), Ge et al. (2021)) to classify the species within this large genus. However, no similar species could be grouped with *I.longulus* sp. nov. Therefore, we are only able to provide a description of the new species, but not able to define any species group at the moment.

## Supplementary Material

XML Treatment for Ichthyurus longulus

XML Treatment for Ichthyurus bourgeoisi

XML Treatment for Ichthyurus davidi

XML Treatment for Ichthyurus pieli

XML Treatment for Ichthyurus savioi

XML Treatment for Ichthyurus senensis

XML Treatment for Ichthyurus vandepolli

XML Treatment for Ichthyurus longulus
